# Tocopherol biosynthesis in *Leishmania* (*L*.) *amazonensis* promastigotes

**DOI:** 10.1002/2211-5463.12613

**Published:** 2019-03-05

**Authors:** José Mário F. Balanco, Rodrigo A.C. Sussmann, Ignasi B. Verdaguer, Heloisa B. Gabriel, Emilia A. Kimura, Alejandro M. Katzin

**Affiliations:** ^1^ Department of Parasitology Institute of Biomedical Sciences University of São Paulo Brazil

**Keywords:** isoprenoid, *Leishmania* (*L*.) *amazonensis*, nitisinone, tocopherol, trypanosomatid, usnic acid

## Abstract

Leishmaniasis is a neglected disease caused by a trypanosomatid protozoan of the genus *Leishmania*. Most drugs used to treat leishmaniasis are highly toxic, and the emergence of drug‐resistant strains has been observed. Therefore, new therapeutic targets against leishmaniasis are required. Several isoprenoid compounds, including dolichols or ubiquinones, have been shown to be important for cell viability and proliferation in various trypanosomatid species. Here, we detected the biosynthesis of tocopherol in *Leishmania* (*L*.) *amazonensis* promastigotes *in vitro* through metabolic labelling with [1‐(n)‐3H]‐phytol. Subsequently, we confirmed the presence of vitamin E in the parasite by gas chromatography–mass spectrometry. Treatment with usnic acid or nitisinone, inhibitors of precursors of vitamin E synthesis, inhibited growth of the parasite in a concentration‐dependent manner. This study provides the first evidence of tocopherol biosynthesis in a trypanosomatid and suggests that inhibitors of the enzyme 4‐hydroxyphenylpyruvate dioxygenase may be suitable for use as antileishmanial compounds.

**Database:**

The amino acid sequence of a conserved hypothetical protein [Leishmania mexicana MHOM/GT/2001/U1103] has been deposited in GenBank (CBZ28005.1)

**Abbreviations**
[3H]‐Phytol: radioactive isotope of hydrogen (tritium)‐phytol
BSTFA: N,O‐Bis(trimethylsilyl) trifluoroacetamide
DAD: diode array detector
DMSO: dimethyl sulfoxide
FBS: fetal bovine serum
GC‐MS: gas chromatography–mass spectrometry
HGA: head group aromatic
HPLC: high‐performance liquid chromatography
HPP: 4‐hydroxyphenylpyruvate
HPPD: 4‐hydroxyphenylpyruvate dioxygenase
HPT: homogentisate prenyltransferase
*L. amazonensis*: *Leishmania* (*Leishmania*) *amazonensis* LV79 (MPRO/BR/72/M1841)
MPBQ: 2‐methyl‐6‐phytylplastoquinol
MTT: (3‐(4,5‐dimethylthiazol‐2‐yl)‐2,5‐diphenyltetrazolium bromide)
NTBC: nitisinone
PBS: phosphate‐buffered saline
Phytyl‐P: phytyl‐phosphate
Phytyl‐PP: phytyl diphosphate
ROS: reactive oxygen species
RP‐HPLC: reversed‐phase high‐performance liquid chromatography
St: staurosporine
TMCS: chlorotrimethylsilane
UA: usnic acid
UV‐Vis: ultraviolet–visible spectroscopy

Human leishmaniasis is a neglected protozoal disease that occurs mostly in tropical and subtropical regions, caused by a protozoan of the genus *Leishmania* and transmitted by the bite of the female sandfly. It affects either the skin or the internal organs and is estimated that 1.5–2 million of new cases occur annually, with approximately 350 million people currently at risk of acquiring the disease 1.

The drugs that have been used most frequently to treat leishmaniasis are the pentavalent antimonials, sodium stibogluconate and meglumine antimoniate; however, these drugs are considerably toxic and, in some areas, the emergence of resistant strains has been observed 2. Therefore, the need for studies on new therapeutic targets against leishmaniasis, such as metabolic pathways, has become extremely urgent. Isoprenoids are very diverse and constitute an abundantly present group of natural products 3. Several essential trypanosomatid metabolites require isoprene biosynthesis by the classical mevalonate pathway located in mitochondria or a leucine alternative pathway in *Leishmania mexicana* 4, 5, 6. Several isoprenoid compounds such as dolichols or ubiquinones have been already shown to be important for cell viability and proliferation in various trypanosomatids 7, 8, 9. The combination of inhibitors that act at various points of the isoprenoid pathway seems to be a useful strategy against *Trypanosoma cruzi* and *Leishmania* spp. 7, 8, 9.

Various genes found in trypanosomatids, including *Leishmania* parasites, are homologous to those found in photosynthetic bacteria, and this is why some authors suggest that these organisms may have a photosynthetic ancestor 10. In most photosynthetic organisms, isoprenoid precursors such as geranylgeranyl diphosphate are required for carotenoid, phytyl diphosphate (phytyl‐PP) and isoprenoid quinones biosynthesis 9, 11, 12 among others. Furthermore, phytyl‐PP provides phytyl moieties as a substrate for tocopherol biosynthesis 12. The head group aromatic (HGA) precursor of tocopherol biosynthesis is the homogentisate whose origin is through the tyrosine degradation pathways. The l‐tyrosine is first transformed into 4‐hydroxyphenylpyruvate (HPP) by tyrosine aminotransferase; then, in a second step, 4‐hydroxyphenylpyruvate dioxygenase (HPPD) catalyses the formation of homogentisate from HPP. HGA is then subject to prenylation with phytyl diphosphate to yield 2‐methyl‐6‐phytylplastoquinol (MPBQ) by the enzyme homogentisate prenyltransferase (HPT). In biosynthesis are ring methylations and ring cyclization. Tocopherol cyclase converts MPBQ to δ*‐* and γ‐tocopherol; finally, tocopherol methyltransferase adds a methyl group to the sixth position of the chromanol ring converting δ‐ and γ‐tocopherol to β‐ and α‐tocopherol 13, 14.

Tyrosine degradation has been previously demonstrated in *Leishmania*, and it appears to be an excellent drug target 15. The degradation starts when the enzyme tyrosine aminotransferase cleaves the amino group of the amino acid. Subsequently, the deaminated tyrosine is reduced by the 4‐hydroxyphenylpyruvate dioxygenase (EC:1.13.11.27), whose activity has been already detected in leishmanial extracts 15. Thus, vitamin E or tocopherol biosynthesis is an attractive target for investigation because *Leishmania* lives in a pro‐oxidant environment in the vertebrate host and must therefore have an efficient antioxidant defence in order to ensure its survival 16. Vitamin E acts as an antioxidant and free radical scavenger and, together with other antioxidants, has a role in the defence against oxidative stress 17. In cultures of embryonic neurons, staurosporine treatment, an oxidative stress and cell death inducer, tocopherol protected the neurons from oxidative damage 18. In plants, tocopherol and tocochromanols protect thylakoid components from oxidative damage and play roles in electron transport reactions, cell membrane permeability and fluidity, thereby acting as membrane stabilizers 19. Previously, tocopherol biosynthesis, its antioxidant function and this biosynthesis inhibited by usnic acid were demonstrated in *Plasmodium falciparum* 20, 21. Here, we reported, for the first time, biochemical evidence indicating that a pathway for vitamin E biosynthesis in *Leishmania* (*L*.) *amazonensis* promastigotes is active. The results also indicate that this biosynthesis can be strongly inhibited by potential HPPD inhibitors.

## Materials and methods

### Reagents

Radiolabelled precursor ([1‐(n)‐^3^H]‐phytol; 20 Ci·mmol^−1^; 1 mCi·mL^−1^) was obtained from Amersham‐Pharmacia Biotech (Buckinghamshire, UK). All solvents used were HPLC grade or higher, chlorotrimethylsilane (TMCS), N,O‐Bis(trimethylsilyl) trifluoroacetamide (BSTFA), MTT (3‐(4,5‐dimethylthiazol‐2‐yl)‐2,5‐diphenyltetrazolium bromide), α‐ and γ‐tocopherols, usnic acid, nitisinone and staurosporine (St) were purchased from Sigma (Darmstadt, Germany), and SYBR Green I and CellROX^®^ Oxidative Stress Reagents from Molecular Probes (Eugene, OR, USA).

### Culture of *Leishmania* (*L*.) *amazonensis* promastigotes

*Leishmania* (*L*.) *amazonensis* LV79 (MPRO/BR/72/M1841) (*L. amazonensis*) was used. The parasites were maintained by sequential passages *in vitro*. For *in vitro* growth, promastigotes were incubated at 25 °C in tissue culture flasks containing tocopherol‐free TC‐100 medium (Vitrocell local^®^) completed with 5% heat‐inactivated fetal bovine serum (FBS) (Vitrocell) (complete TC‐100 medium) with passages every seven days. Control tests to avoid culture contamination were performed continuously.

### MTT cell proliferation assay

The MTT assay, adapted for *Leishmania* spp. 22, was used to assess promastigote viability in different experiments. Briefly, promastigotes were pelleted by centrifugation and washed twice in PBS (137 mm NaCl, 2.7 mm KCl, 10 mm Na_2_HPO_4_ and 1.8 mm KH_2_PO_4_, pH 7.4). Finally, parasites were resuspended in PBS, to which 5.0 mg·mL^−1^ of MTT was added. After incubation for 60 min at 25 °C, 0.1 mL of a 10% of SDS lysis solution was added to stop the reaction 22. Absorbance was monitored using a fluorometer POLARstar^®^ Omega (BMG Labtech, Waltham, MA, USA), at 595 nm and 690 nm as reference. The MTT methodology was used to perform three kinds of experiments as described below.

We studied the antileishmanial activity of two HPPD inhibitors. Promastigotes in late logarithmic phase were treated with various concentrations (2.5 μm to 0.078 μm) of usnic acid (UA) or nitisinone (NTBC) (250 μm to 3.9 μm), and parasite viability was evaluated by MTT assay. Control cultures were treated with DMSO and/or acetone as solvent controls, and their effects on parasite viability were assessed daily by MTT measurements as well. The inhibitor concentration where the growth is reduced by half (IC_50_) was calculated after 72 h of treatment.

In the second step, we studied whether tocopherol could recover HPPD inhibitor effects on parasite viability. Using a concentration of usnic acid or nitisinone where the growth is reduced near by half, promastigotes in late logarithmic phase were treated for 72 h with 0.5 μm usnic acid or for 48 h with 31.25 μm NTBC with or without exogenously added 25 μm α‐tocopherol in the complete medium and the parasite viability was evaluated by MTT methodology.

In the third step, we studied staurosporine and tocopherol interactions. Promastigotes in late logarithmic phase were treated with staurosporine 75 nm in TC‐100 medium with or without various concentrations of α‐tocopherol (30, 60 or 90 μm). We previously observed that the described staurosporine concentrations and periods of treatment produce viability decreases. After 1‐h incubation at 25 °C, the cells were washed twice and the culture was recovered in complete TC‐100 medium. Then, the treatments effects on parasite viability were assessed by MTT measurements.

### Metabolic labelling and tocopherol inhibition assays

Cultures of *L. amazonensis* promastigotes, mainly in late logarithmic phase, were labelled with [1‐(n)‐^3^H]‐phytol (3.125 μCi·mL^−1^) in complete TC‐100 medium for 18–24 h and were then recovered. Subsequently, the parasite cultures were centrifuged at 800 ***g*** for 10 min at 4 °C. The parasites were purified as described above, and each was subjected to vitamin E extraction.

For tocopherol inhibition assays, similar amounts of promastigotes, primarily in late logarithmic phase, were treated or not with 0.25 μm usnic acid for 72 h or 31.25 μm nitisinone for 48 h. The parasites were incubated with the radiolabelled precursor [^3^H]‐phytol (0.75 μCi·mL^−1^) for the last 18–24 h before starting the extraction procedure. Biosynthesis of tocopherols of the same amount of treated and untreated parasites was compared by metabolic labelling and RP‐HPLC (system I) as described below.

### Vitamin E extraction

A 3 mL volume of 0.2 M HClO_4_ in cool methanol was added to 5–10 × 10^8^ promastigote pellets lyophilized in glass tubes. After mixing for 1 minute in a vortex, 3 mL of petroleum ether (bp 60–80 °C) was added 3 times. The sample was mixed for 1 minute on a vortex and centrifuged at 2700 ***g*** for 20 minutes at 4 °C. The petroleum ether phase was collected, and the extraction procedure was repeated again twice. The supernatants were transferred to glass tubes and evaporated in a stream of nitrogen at room temperature. The residues were resuspended in 400 μL of each HPLC initial mobile phase (see below) and subjected to RP‐HPLC 23, 24 and/or mass spectrometry analysis 25.

### Reversed‐phase high‐performance liquid chromatography

Two reversed‐phase high‐performance liquid chromatography (RP‐HPLC) systems (see below) were standardized in order to resolve satisfactorily the investigated isoprenoid compounds in *L. amazonensis* labelled with [^3^H]‐phytol. We determined the retention time of isoprenoid compounds as α‐ and γ‐tocopherol monitoring at 295 nm. The isoprenoid compounds α‐ and γ‐tocopherols were co‐injected as internal standards with the extracts of labelled parasites to assess their retention time. In experiments employing metabolically labelled precursors, each fraction was collected per minute. The subsequent fractions were dried, resuspended in 600 μL of scintillation liquid mixture (PerkinElmer Life Sciences, MA, USA) and counted with a Beckman 5000 β‐radiation scintillation counter^®^ (Beckman, CA, USA). The stationary phase was a Phenomenex Luna^®^ C18 column (250 mm × 4.6 mm × 5 um) (Phenomenex, Torrance, CA, USA) coupled to pre‐C18 column (Phenomenex), detector UV‐Vis or a diode array detector (DAD) (Gilson 170) and a fraction collector FC203B. For data processing, we used trilution ^™^ (Middleton, WI, USA) LC 3.0 System Software.

System I: The mobile phase consisted of a linear gradient system between acetonitrile (A) and methanol (B). The initial condition was 50% (A) at 0 min, reaching 70% (A) at 28 min and maintained for 10 min longer. The proportion returned to 50% (A) at 40 min with a flow rate of 1 mL·min^−1^, and the fractions were collected per minute.

System II: The isocratic mobile phase consisted of methanol/ethanol 50:50 (v/v) with a flow rate of 1 mL·min^−1^. Fractions were collected per minute.

### Gas chromatography–mass spectrometry

To investigate the presence of α‐tocopherol, the fractions resulting from RT‐HPLC (System I) of *L. amazonensis* extracts were analysed by gas chromatography–mass spectrometry (GC‐MS) to confirm the presence of α‐tocopherol and derivatized as described by Van Pelt, 1998 23. We used a Y2K ion trap mass spectrometer (MS) PolarisQ System^®^ (Finnigan, ThermoQuest Inc., San Jose, CA) equipped with a nanosource type electron impact ionization (EI) and coupled with a TRACE GC^®^ (Finnigan, ThermoQuest Inc., San Jose, CA) equipped with a 30 m × 25 mm × 0.25 μm DB‐5‐ms column. The mobile phase was helium at a flow rate of 1 mL·min^−1^.

The GC‐MS analysis conditions were an initial oven temperature of 120 °C for 2 minutes followed by a ramp of 20 °C·min^−1^ until 320 °C. This temperature was maintained for 3 minutes and then cooled to initial conditions. The injector temperature was 230 °C, and the transfer line temperature was 295 °C. For MS analysis, the temperature of ion source was 200 °C, and two segments were acquired, first monitoring a range of *m/z* 150 to 505 (full scan) and second monitoring the fragmentation (MS/MS) of ion at *m/z* 502 corresponding to the derivatized α‐tocopherol. The excitation energy scale for fragmentation was 0.3 and 1 V. The monitored fragment ions of α‐tocopherol were at *m/z* 236, 237 and 277. The mass spectra were analysed using the version 1.3 xcalibur ^®^ (Waltham, MA, USA) data analysis program.

### Reactive oxygen species measurement

Reactive oxygen species (ROS) production was measured using CellROX^®^ Oxidative Stress Reagent fluorogenic probes to measure ROS in live cells 26. The untreated promastigotes (negative control) are treated with UA 0.5 μm for 96 h with or without 25 μm α‐tocopherol, washed with PBS and loaded with 5 μm of the CellROX Green Reagent in PBS for 30 min at 25 °C. Fluorescence was monitored using a fluorometer POLARstar^®^ Omega (BMG Labtech), at 485 nm and 520 nm for excitation and emission, respectively.

### Statistical analysis

Statistical significance was determined by Student's t‐test or ANOVA using graphpad prism ^®^ Software (GraphPad Software, Inc., La Jolla, CA, USA). The obtained *P*‐values are indicated throughout Results section. Parasite growth inhibition assays were also analysed using graphpad prism ^®^ Software. IC_50_ values were estimated fitting the data to a nonlinear regression (dose–response slope/variable sigmoid equation). We only considered as dose–response functions those assays with an R‐squared values ≥ 0.95. All experiments were performed in three independent biological assays and each one in three or two independent technical replicates as indicated for each one experiment.

## Results

### Biosynthesis of vitamin E

**Figure 1:**
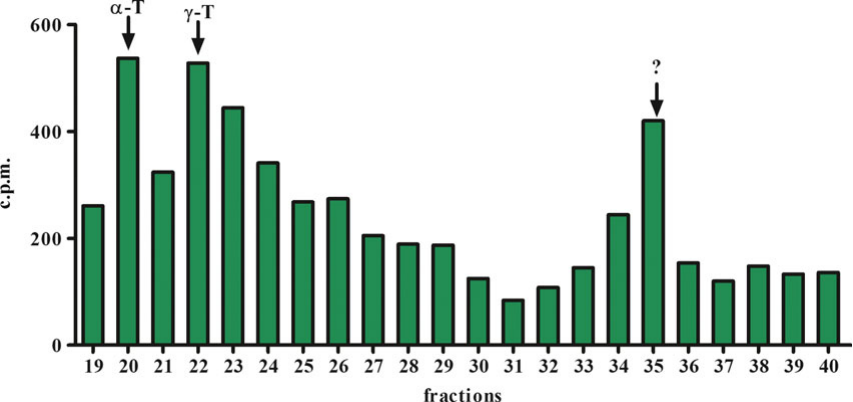
Radioactive elution profile employing radiolabelled precursor and/or RP‐HPLC system. Profile of the promastigotes of *Leishmania* (*L*.) *amazonensis,* metabolically labelled with [1‐(n)‐^3^H]‐phytol. Extracts from promastigotes stages were purified by RP‐HPLC. Fractions were collected at intervals of 1 mL·min^−1^. The retention time of compounds was identified by co‐injection of commercial standards in both systems. α‐T: α‐tocopherol, γ‐T: γ‐tocopherol. ?: unknown. Three independent experiments. c.p.m., count per minute.

The γ‐ and α‐tocopherols biosynthesized by *L. amazonensis* promastigotes were identified by two RP‐HPLC systems. The parasite labelled with [1‐(n)‐^3^H]‐phytol was analysed by the RP‐HPLC (system I) and a radioactive fraction with a coincident retention time with α‐tocopherol (20 and 21 min) and γ‐tocopherol (22/23 and 24 min) standards (Fig. 1). Furthermore, a radioactive fraction was detected in the retention time of 34 and 35 minutes (unknown peak). Using another RP‐HPLC system (system II), a radioactive fraction with a coincident retention time with α‐tocopherol standards was also detected in promastigotes (Fig. S1).

### Identification of α‐tocopherol by GS‐MS/MS

**Figure 2:**
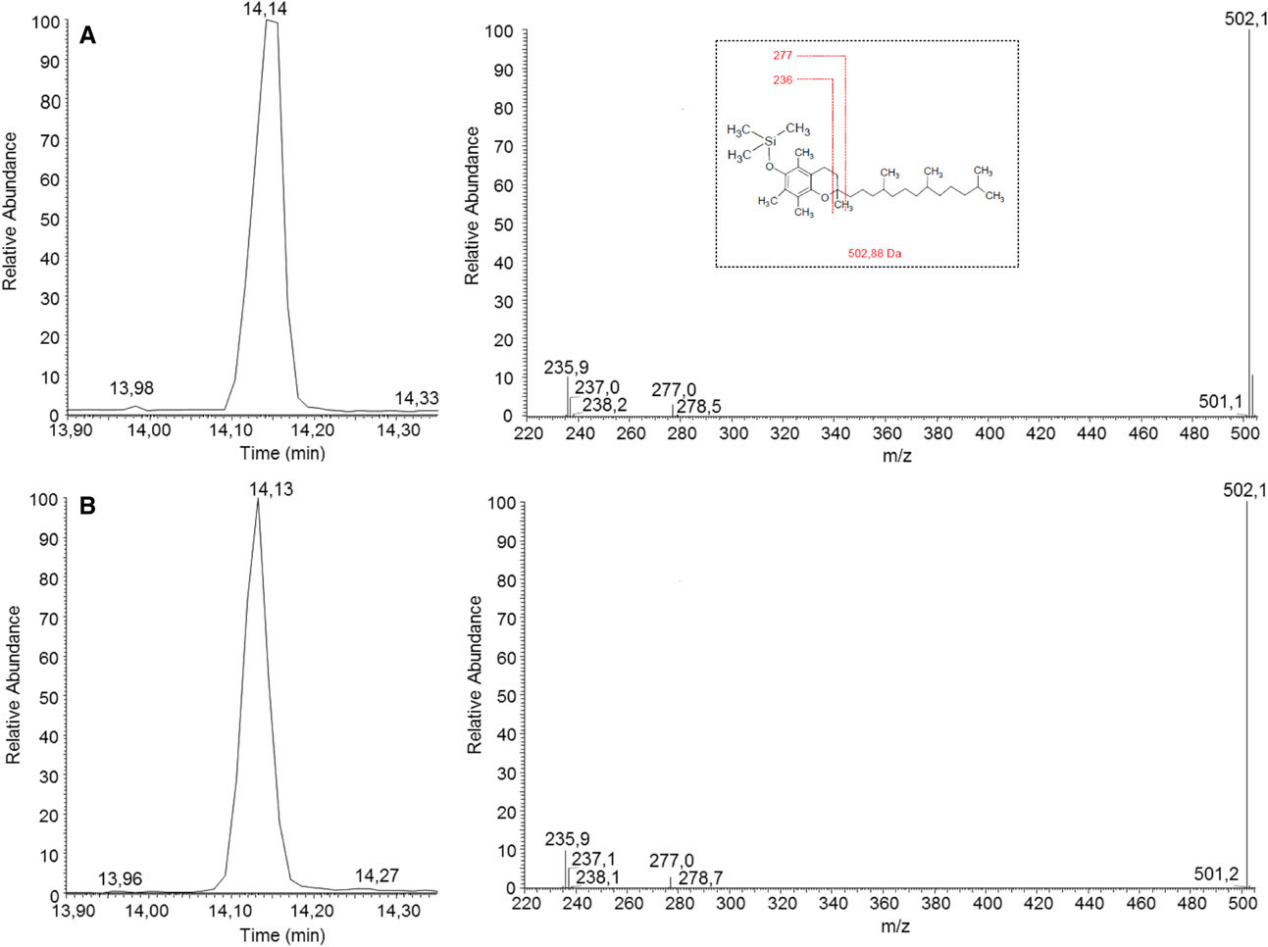
Molecular identification of α‐tocopherol biosynthesis in promastigotes of *Leishmania* (*L*.) *amazonensis* by GC‐MS/MS. Chromatograms and spectra from (A) α‐tocopherol standard; (B) HPLC peaks from 1.25 × 10^10^ parasites. All samples were previously purified by RP‐HPLC. The molecular structure was confirmed by comparing the retention time of GC and the MS2 spectrum of the parental ions at *m/z* 502 for α‐tocopherol.

In addition to metabolic labelling, qualitative GC‐MS/MS analysis was employed for RP‐HPLC fractions of unlabelled promastigotes. Figure 2A shows the chromatogram and fragmentation spectra of the α‐tocopherol standard. The characteristic ions of α‐tocopherol standard are the molecular ion at *m/z* 502 and the fragmentary ions at *m/z* 236 and 277. All were detected in promastigote forms (Fig. 2B). The ion at *m/z* 236 corresponds to the benzene portion with or without a proton, and the fragmentary ion at *m/z* 277 corresponds to the benzene portion plus the cyclized portion of the phytyl side chain.

### Usnic acid and nitisinone effects on vitamin E biosynthesis

**Figure 3:**
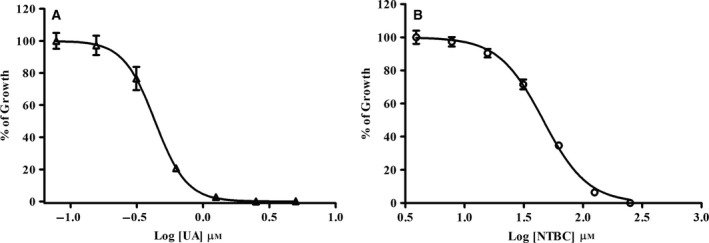
Dose–response curves of parasite viability in the presence of usnic acid (UA) or nitisinone (NTBC) at 72 h of treatment by MTT methodology. (A) Usnic acid inhibited parasite growth in a dose‐dependent manner (IC_50_: 0.433 ± 0.071 μm), and (B) NTBC inhibited parasite growth in a dose‐dependent manner too (IC_50_: 47.87 ± 10 μm). Data are representative of three independent experiments; each experiment done contained three technical triplicates. These data are expressed as means ± SD.

Parasite growth was inhibited in a concentration‐dependent manner with an IC_50_ value of 0.433 μm (± 0.071) of usnic acid (Fig. 3A) and an IC_50_ value of 47.87 μm (± 10) of nitisinone (Fig. 3B) at 72 h of treatment.

**Figure 4:**
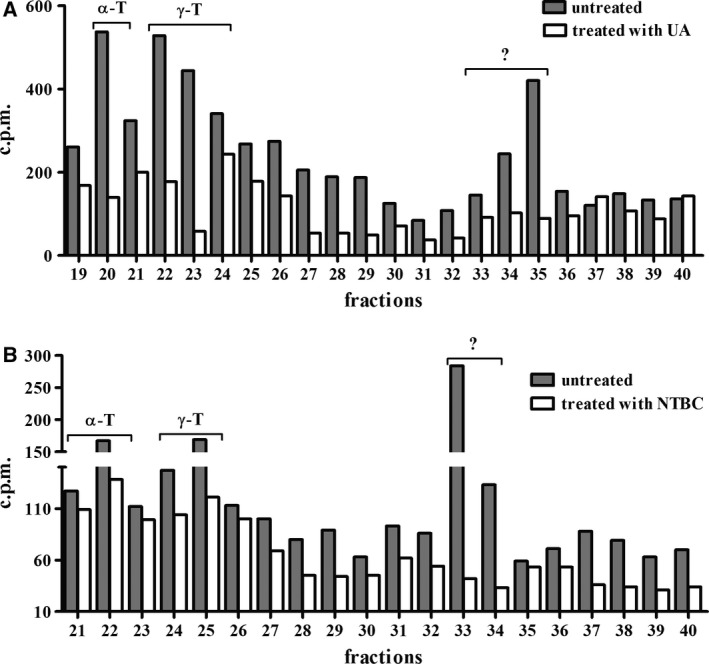
Inhibition of vitamin E biosynthesis in parasites treated with UA or NTBC for *in vitro* cultures of the *Leishmania* (*L*.) *amazonensis* promastigotes. (A) Radioactive elution profile of promastigotes treated (white bars) or untreated (grey bars) for 72 h with UA and labelled with [1‐(n)‐^3^H]‐phytol in the last 24 h. α‐T: α‐tocopherol, γ‐T: γ‐tocopherol, ?: unknown. (B) Radioactive elution profile of promastigotes treated (white bars) or untreated (grey bars) for 48 h with NTBC and labelled with [1‐(n)‐^3^H]‐phytol in the last 24 h. α‐T: α‐tocopherol, γ‐T: γ‐tocopherol, ?: unknown. Data are representative of two independent experiments. c.p.m.: count per minute.

The effects of usnic acid or nitisinone on vitamin E biosynthesis at the promastigote stage were further investigated using a drug concentration and a treatment period that produced a visible but weak decrease in parasite growth. Our results show a significant decrease in α‐tocopherol (59.05 ± 6.42) and γ‐tocopherol (58.18 ± 3.44) biosynthesis in usnic acid‐treated promastigotes (Fig 4A). When promastigotes were treated with nitisinone, the level of inhibition was lower, 24 ± 5.89 for α‐tocopherol and 15.10 ± 1.24 for γ‐tocopherol (Fig. 4B). The most prominent inhibitory effect of nonspecific inhibitor usnic acid, decreasing almost all fractions, may indicate a general effect on parasite metabolism.

**Figure 5:**
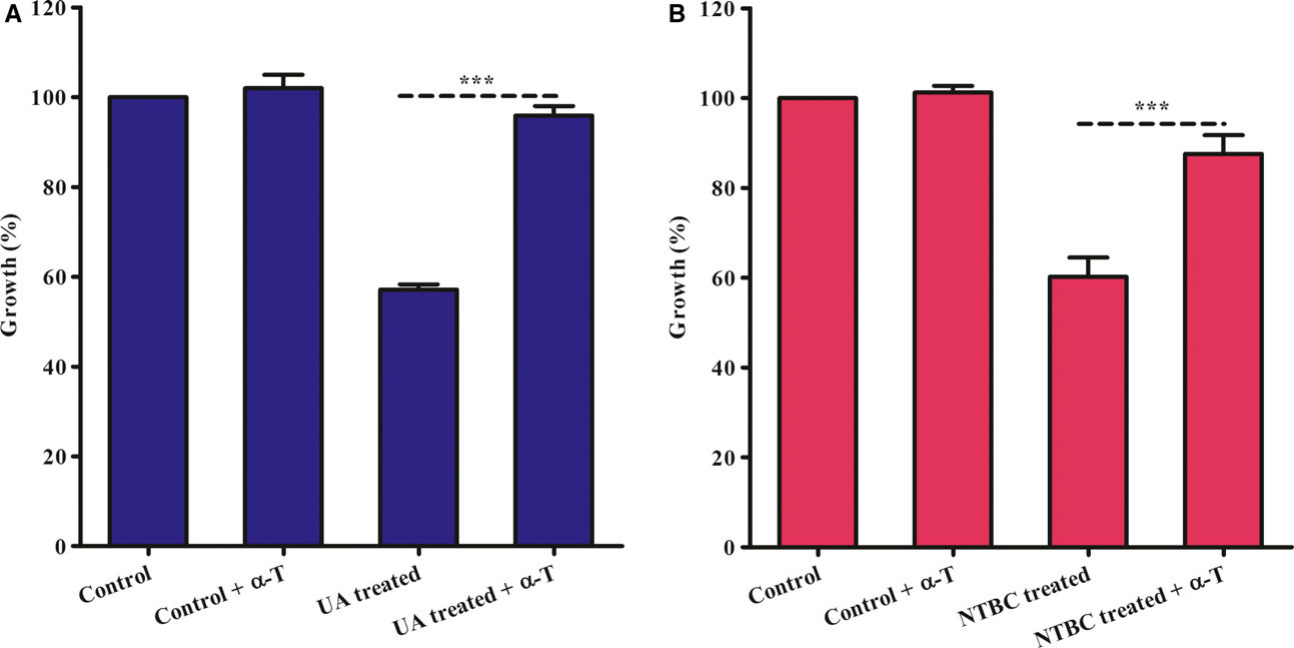
Effect of α‐tocopherol exogenous in recover of growth of promastigotes treated with UA or NTBC. (A) Recovery of parasites treated for 72 h at 25 °C with UA by exogenously added α‐tocopherol. (Control) parasites control; (control + α‐T) parasites control plus α‐tocopherol; (UA treated) parasites treated with 0.5 μm of UA; (UA treated plus α‐T) parasites treated with 0.5 μm of UA plus 25 μm of α‐tocopherol. (B) Recovery of parasites treated for 48 h at 25 °C with NTBC by α‐tocopherol exogenous. (Control) parasites control; (control + α‐T) parasites control plus α‐tocopherol; (NTBC treated) parasites treated with 31.25 μm of NTBC; (NTBC treated + α‐T) parasites treated with 31.25 μm of NTBC plus 200 μm α‐tocopherol. O.D., optical density. MTT for 1 h at 25 °C. (***) *P* < 0.0001 ANOVA. Data are representative of three independent experiments; each experiment done contained three technical triplicates. These data are expressed as means ± SD.

**Figure 6:**
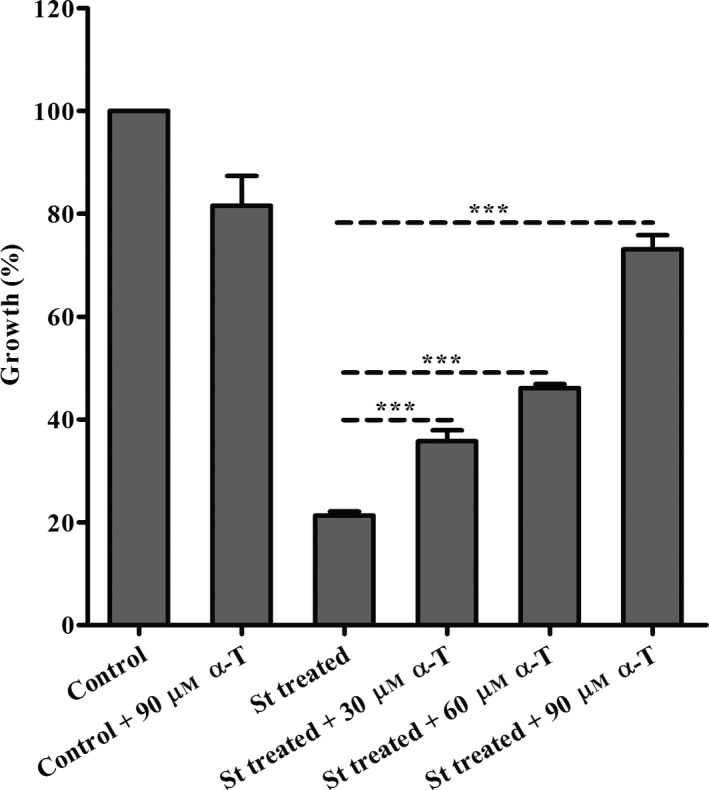
Recovery test of *Leishmania* (*L*.) *amazonensis* promastigotes treated with staurosporine (St) and α‐tocopherol (α‐T) at 25 °C for 72 h. (Control) parasites control; (control + 90 μm α‐T) parasites control plus 90 μm of α‐tocopherol; (St treated) parasites treated with 75 nm staurosporine; (St treated + 30 μm α‐T) parasites treated with 75 nm staurosporine plus 30 μm α‐tocopherol; (St treated + 60 μm α‐T) 75 nm staurosporine plus 60 μm α‐tocopherol; (St treated + 90 μm α‐T) parasites treated with 75 nm staurosporine plus 90 μm α‐tocopherol. Viability by MTT assay for 1 h at 25 °C. OD, optical density. (***) *P* < 0.0001 ANOVA. Data are representative of three independent experiments; each experiment done contained three technical triplicates. These data are expressed as means ± SD.

We attempted to reverse this growth inhibition by usnic acid and nitisinone by the exogenous addition of α‐tocopherol compared with parasites treated only with tocopherol. Our results show that exogenously added α‐tocopherol partially reversed growth inhibition in a dose‐dependent manner (Fig. 5A,B). This finding suggests the vitamin E biosynthesis in the parasite and that usnic acid or nitisinone may affect it. Moreover also, the exogenous addition of α‐tocopherol protects the parasites treated with 75 nm of staurosporine (st) (an inhibitor of protein kinases that induces ROS production and cell death) 18, reversed partially the viability of the parasites in a dose‐dependent manner (Fig. 6).

### The function of vitamin E in *Leishmania* (*L*.) *amazonensis*

**Figure 7:**
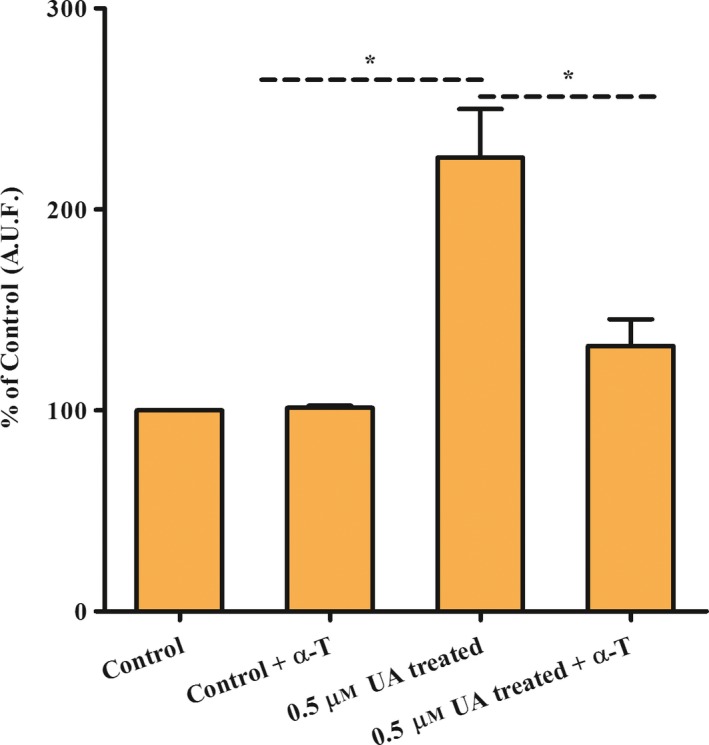
Reactive oxygen species (ROS) measurement in *Leishmania* (*L*.) *amazonensis* promastigotes UA treated for 96 h. CellROX assay I. (Control) parasites control; (control + α‐T) parasites plus α‐tocopherol; (0.5 μm UA treated) parasites treated with 0.5 μm of usnic acid and (0.5 μm UA treated + α‐T) parasites treated with 0.5 μm of usnic acid plus 25 μm of α‐tocopherol. ROS was estimated by the AUF (arbitrary unit of fluorescence). (*) *P* < 0.005 ANOVA. Data are representative of three independent experiments; each experiment done contained two technical triplicates. These data are expressed as means ± SD.

To assess whether vitamin E biosynthesis in *L. amazonensis* parasites acts against oxidative stress, we measured intracellular ROS levels in parasites treated with usnic acid, the HPPD inhibitor that showed the strongest tocopherol inhibitory activity. Usnic acid treatment increased intracellular ROS content, compared to untreated promastigotes. However, we still do not know whether the principal usnic acid ROS effects are due to tocopherol biosynthesis inhibition or whether other mechanisms are also involved. Moreover, this effect was reversed when exogenously α‐tocopherol was supplemented in the culture medium (Fig. 7).

### Predicted amino acid sequences of conserved hypothetical proteins in *Leishmania mexicana* and *Leishmania donovani*

On BLAST analysis, we found the LMXM 26_1830 conserved putative protein in the genome of *L. mexicana* with closest similarity of 4‐hydroxyphenylpyruvate dioxygenase superfamily and LMXM_28_1320 putative prenyltransferase protein of *L. mexicana* and LDBPK_28_1430 putative prenyltransferase protein of *Leishmania donovani*. Using the program T‐COFFEE WS, the alignment of *Arabidopsis thaliana* HPPD with the predicted amino acid sequence of LMXM26_1830 showed the conserved regions with amino acid residues with similarity of HPPD (Fig. S2), and using *Chlamydomonas reinhardtii* homogentisate phytyltransferase (EC:2.5.1.115) (HPT1) as a query, with predicted amino acid sequence of LMXM_28_1320 putative prenyltransferase protein of *L. mexicana* and LDBPK_28_1430 putative prenyltransferase protein of *L. donovani*, it showed the conserved prenyl‐DP and divalent cation‐binding domains in the N‐terminal region in similarity of HPT1 27.

The alignment of putative prenyltransferase of *L. mexicana* and *L. donovani* with three HPT1 from different origins, *Arabidopsis thaliana* HPT1, *Synechocystis* sp. PCC 6714 HPT1 and *Chlamydomonas reinhardtii* HPT1, showed a conserved amino acid residues for catalytic activity in haem‐O‐synthase, which are conserved in HPT1 27 (Fig. S3).

## Discussion

The present study showed for the first time that the parasite *L. amazonensis* promastigotes biosynthesizes vitamin E. Although vitamin E biosynthesis is characteristic of photosynthetic eukaryotes 28, it was also demonstrated in *P. falciparum* 20, 21. In *L. amazonensis*, using two different RP‐HPLC systems and the metabolic precursor [1‐ (n) ‐3H]‐phytol, two fractions with the same retention time as the α‐ and γ‐tocopherol standards were detected in the promastigotes. The eluted compound identities, studied by GC‐MS/MS, indicated the presence of α‐tocopherol isomers. Moreover, tocopherols were not detectable in complete culture medium, reinforcing the findings of tocopherol biosynthetic pathway in the parasite (data not shown). The presence of γ‐tocopherol in promastigotes provides further evidence*,* since γ‐tocopherol is an intermediate compound for the formation of α‐tocopherol as shown in *Arabidopsis* spp. 29 and *Synechocystis* spp. 30 among other photosynthetic organisms. In *Arabidopsis* spp., phytol released from chlorophyll can be converted into phytyl‐P and phytyl‐PP and subsequently employed for tocopherol synthesis through a salvage pathway 31. Radiolabelled phytol incorporation into tocopherol also demonstrates that the parasite must possess a salvage pathway as does *Arabidopsis* spp., by incorporating and recycling phytol for tocopherol isoprenylation among other phytylated compounds. *Leishmania donovani* and photosynthetic green sulfur bacteria remain the only known organisms that biosynthesize chlorobiumquinone, an isoprenoid naphthoquinone containing carbonyl group in the isoprenoid side chain. This compound is thought to prevent oxidative stress derived from the parasite–host relationship 32. Chlorobiumquinone and tocopherol biosynthesis in various *Leishmania* species may be considered a trypanosomatid photosynthetic ancestor. This theory is also supported by genetic studies based on gene homology 32.

Our results also indicate that this biosynthesis can be strongly inhibited by the HPPD irreversible enzyme‐binding inhibitor usnic acid 20, 21, 33 and moderately by HPPD inhibitor nitisinone 34, 35, 36, possibly due to its reversible enzyme‐binding in most studied organisms 37, 38, 39. The 4‐hydroxyphenylpyruvate dioxygenase catalyses the conversion of *p*‐hydroxyphenylpyruvic acid to homogentisic acid 37, a direct precursor of vitamin E and plastoquinone in most photosynthetic organisms 38 except in cyanobacteria, where plastoquinone is thought to be biosynthesized by an alternative pathway 39. Therefore, inhibition of 4‐hydroxyphenylpyruvate dioxygenase enzyme decreases vitamin E and plastoquinone biosynthesis in most photosynthetic organisms 38, 39. Because plastoquinone is a required cofactor for phytoene desaturation, its inhibition also produces a carotenoid biosynthesis decrease in most photosynthetic organisms 38, 39. Norris *et al*. and Dahnhardt 38, 39 showed that vitamin E but not plastoquinone biosynthesis is disrupted in mutants that lack the encoding gene of HPPD, proving its importance for compound formation.

4‐hydroxyphenylpyruvate dioxygenase may be considered a ‘double‐edged sword’, because it has effects at various *Leishmania* metabolic steps as well in several blood‐feeding arthropods 15, 40. Tyrosine degradation has already been demonstrated to be necessary for *Leishmania* parasites, and dehydrogenase activity for p‐hydroxybenzoate degradation has been previously detected in parasite extracts 15. Tyrosine degradation appears to be an excellent drug target against *Leishmania* 15; therefore, HPPD inhibitors may also act at this point. Surprisingly, tyrosine catabolism appears to be an essential treat to blood‐feeding arthropods. Nitisinone is a commercially available low‐toxicity compound that has been already investigated as a selective insecticide; it has an ability to hinder transmission of blood‐feeding arthropod‐dependent infectious diseases 40.

*Leishmania* (*L*.) *amazonensis* showed growth inhibition upon drug treatment with usnic acid or nitisinone. Moreover, this inhibition was partially reversed by the addition of α‐tocopherol, suggesting that vitamin E biosynthesis is commonly present by the *Leishmania* parasite and that HPPD inhibitors may specifically affect this biosynthetic pathway. The strong decrease in vitamin E biosynthesis when parasites were treated with usnic acid and the weak decrease following nitisinone treatment suggest an inhibitory activity on 4‐hydroxyphenylpyruvate dioxygenase as described by Meazza *et al*. 41, Romagni *et al*. 33, Ellis *et al*. 34 and Laschi *et al*. 35 in other cell systems. The antileishmanial activity of usnic acid has been previously tested in *L. amazonensis in vivo*. Intralesional administration was observed at a significant effect that reduced the weight of lesions and parasite loads in infected footpads 42 in *T. cruzi* cultivated *in vitro* 43. The parasites of *L. amazonensis* live in a pro‐oxidant environment and therefore have evolved extensive detoxifying and protective mechanisms against ROS 16. Nevertheless, tocopherol can efficiently scavenge various radicals released during parasite oxidative stress 44.

Staurosporine is an inhibitor of protein kinases, and its growth inhibitory effects in the parasite were also partially reversed in a dose‐dependent manner (Fig. 6). Staurosporine rescue suggests that tocopherol may protect against glutathione depletion and staurosporine‐induced oxidative stress and cell death as it happens in other cell systems; nevertheless, further studies are needed to better understand this phenomenon 18, 45, 46. Usnic acid treatment produced important effects on ROS production that can be protected by tocopherol. These findings suggest that tocopherol may be involved in parasitic usnic acid‐induced oxidative stress defence; however, we still do not know whether usnic acid‐induced effects on ROS levels occur because of tocopherol biosynthesis inhibition or whether other mechanisms are involved as well. Similar results regarding tocopherol functions were previously investigated in other cell systems. For example, in *P. falciparum* biochemical studies confirmed vitamin E biosynthesis, which can be inhibited by usnic acid, and that the vitamin E produced endogenously is active as an antioxidant 20, 21. A study involving the *Euglena gracilis* W_14_ZUL strain found that an organic carbon source resulted in increased mitochondrial activity, ROS and tocopherol biosynthesis 47. Another group showed increased vitamin E biosynthesis in *Rosmarinus officinalis L*. under water stress that was related to limited photosynthesis and higher oxygen formation 48. *Synechocystis* spp. tocopherol‐deficient mutants were challenged with various combinations of chemicals and/or abiotic stresses to induce formation of various ROS types. The study showed that, compared with the wild‐type, mutants of *Synechocystis* spp. challenged with linolenic acid and high luminosity accumulated a higher level of lipid peroxides 49.

In conclusion, more experiments should be performed to conclude that promastigotes of *L. amazonensis* have an active pathway for tocopherol biosynthesis as well as an active phytol metabolism. Ultrastructural, biochemical and biological analyses are required to elucidate completely the physiologic role of tocopherol in *L. amazonensis*, as well as its biosynthetic pathway. Our experiments suggest that tocopherol may play an important antioxidant role in *L. amazonensis*. Based on these results, a new drug target is proposed for the development of antileishmanial drugs.

## Conflict of interest

The authors declare no conflict of interest.

## Author contributions

AMK, JMFB and RACS conceived and supervised the study. JMFB, RACS HBG and IBV designed experiments. JMFB, RACS HBG and IBV performed experiments. JMFB and EAK provided new tools and reagents. JMFB, RACS, IBV, HBG, EAK and AMK analysed data. JMFB, RACS, IBV and HBG wrote the manuscript. JMFB, RACS, IBV, HBG, EAK and AMK made manuscript revisions.

## Supporting information


**Fig. S1.** Radioactive elution profile employing radiolabelled precursor and/or RP‐HPLC system.
**Fig. S2.** Amino acid sequence alignment of *Arabidopsis thaliana* HPPD with predicted amino acid sequence of LMXM_26_1830 conserved hypothetical protein of *Leishmania mexicana*.
**Fig. S3.** Multiple alignment of amino acid sequence of *Chlamydomonas reinhardtii* HPT1 (accession n° CDZ_92710.1), *Arabidopsis thaliana* HPT1 (accession n° NP_849984.1), *Synechocystis* sp. PCC 6714 HPT1 (accession n° WP_028948637.1) with predicted amino acid sequence of LMXM_28_1320 putative prenyltransferase protein of *Leishmania mexicana* (XP_003876973.1) and LDBPK_28_1430 putative prenyltransferase protein of *Leishmania donovani* (XP_003862242.1).
