# Supplementary material for: Tocopherol biosynthesis in Leishmania (L.) amazonensis promastigotes
Source: FEBS Open Bio. 2019 Mar 5;9(4):743–54. doi: 10.1002/2211-5463.12613 (PMC6443866; doi:10.1002/2211-5463.12613)
Supplement: Supplementary file 1 — Fig. S1. Radioactive elution profile employing radiolabelled precursor and/or RP‐HPLC system. Fig. S2. Amino acid sequence alignment of Arabidopsis thaliana HPPD with predicted amino acid sequence of LMXM_26_1830 conserved hypothetical protein of Leishmania mexicana. Fig. S3. Multiple alignment of amino acid sequence of Chlamydomonas reinhardtii HPT1 (accession n° CDZ_92710.1), Arabidopsis thaliana HPT1 (accession n° NP_849984.1), Synechocystis sp. PCC 6714 HPT1 (accession n° WP_028948637.1) with predicted amino acid sequence of LMXM_28_1320 putative prenyltransferase protein of Leishmania mexicana (XP_003876973.1) and LDBPK_28_1430 putative prenyltransferase protein of Leishmania donovani (XP_003862242.1). [file FEB4-9-743-s001.docx]

**Supporting Information**

**Tocopherol biosynthesis in *Leishmania (L.) amazonensis* promastigotes.**

José Mário F. Balanco, Rodrigo A. C. Sussmann, Ignasi B. Verdaguer, Heloisa B. Gabriel, Emília A. Kimura, Alejandro M. Katzin ^*^.

Department of Parasitology, Institute of Biomedical Sciences, University of São Paulo, São Paulo, Brazil.

**Figure S1. Radioactive elution profile employing radiolabelled precursor and/or RP-HPLC system.** Profile of the promastigotes of *Leishmania (L.) amazonensis,* metabolically labelled with [1-(n)-^3^H]-phytol. Extracts from promastigotes stages were purified by RP-HPLC (Protocol II). Fractions were collected at intervals of 1 ml/min. The retention time of compounds was identified by co-injection of commercial standard. α-T: α-tocopherol. c.p.m.: count per minute.

**Figure S2. Amino acid sequence alignment of *Arabidopsis thaliana* HPPD with predicted amino acid sequence of LMXM_26_1830 conserved hypothetical protein of *Leishmania mexicana*.** The regions with blue colour background show the conserved sequences with high similarity of 4-hydroxyphenylpyruvate dioxygenase and the three amino acid residues the catalytic iron are identified by a red colour asterix. The nucleotide sequence has been deposited in GenBank (accession number: CBZ28005.1).

**Figure S3. Multiple alignment of amino acid sequence of *Chlamydomonas reinhardtii* HPT1 (accession nº CDZ_92710.1), *Arabidopsis thaliana* HPT1 (accession nº NP_849984.1), *Synechocystis* sp. PCC 6714 HPT1 (accession nº WP_028948637.1) with predicted amino acid sequence of LMXM_28_1320 putative prenyltransferase protein of *Leishmania mexicana* (XP_003876973.1) and LDBPK_28_1430 putative prenyltransferase protein of *Leishmania donovani*** (**XP_003862242.1).** The blue colour shading indicates identity in amino acid residues, the conserved prenyl-DP is indicated by black box and the putative catalytic domain is indicated by red box. The amino acids required for catalytic activity in heme-O-synthase, which are conserved in HPT1, are identified by red colour asterix [27].


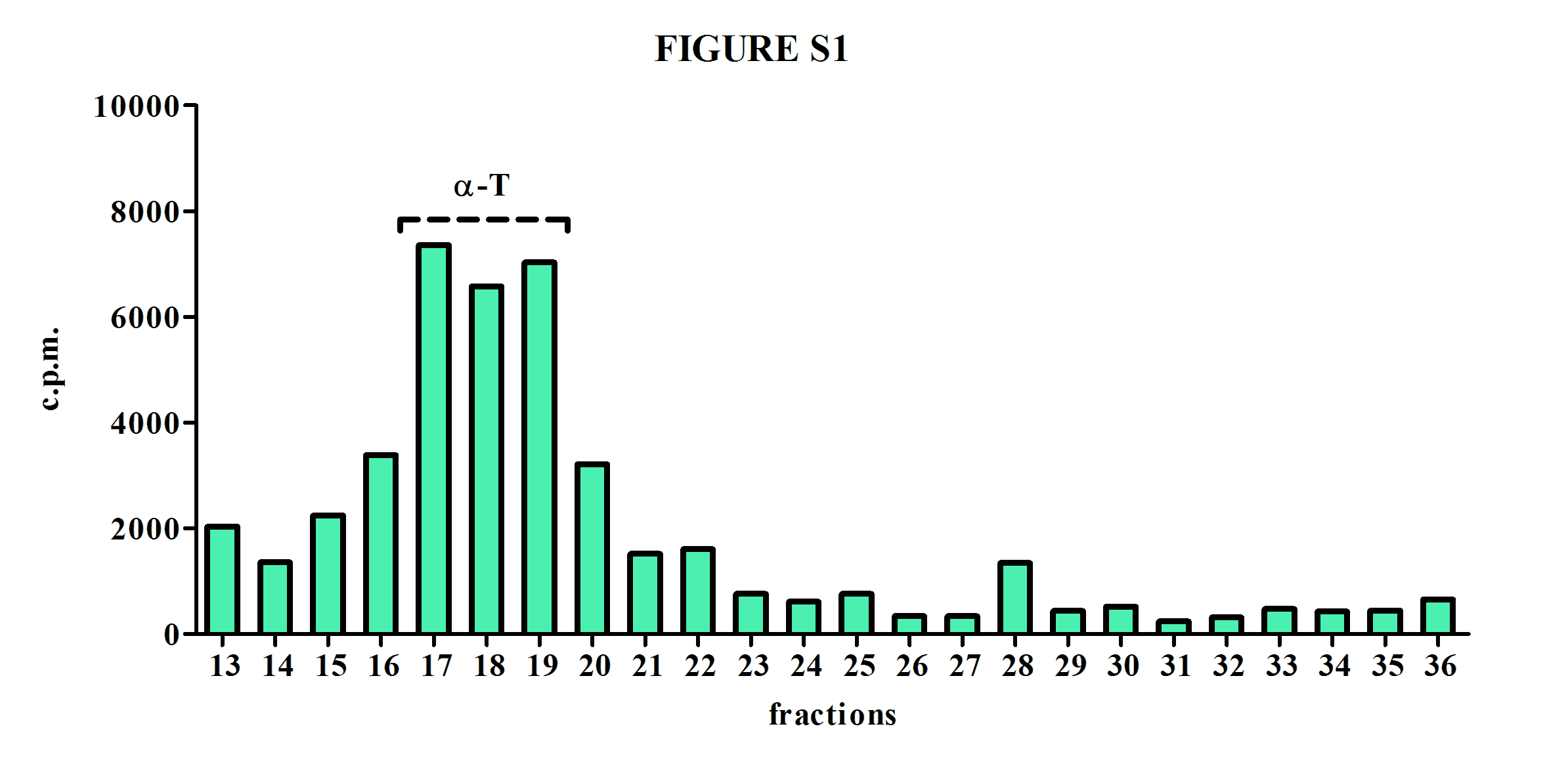


**
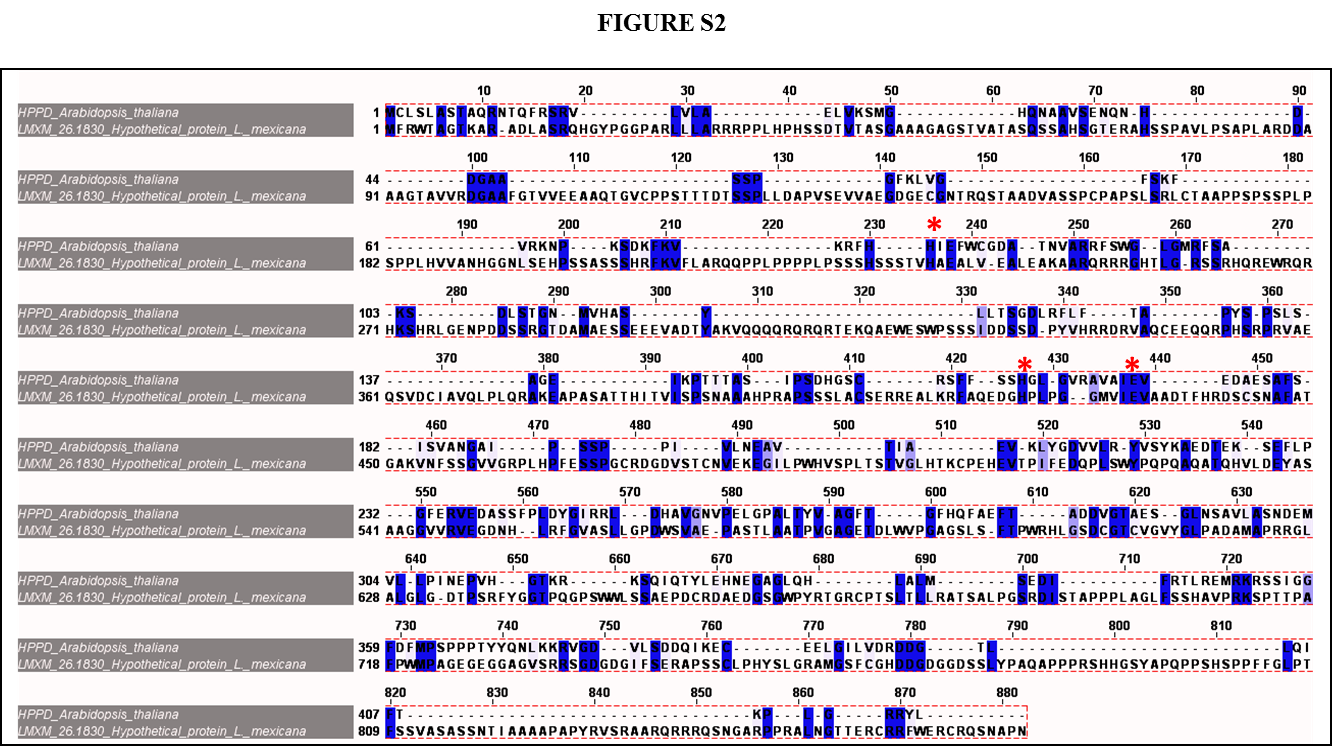
**


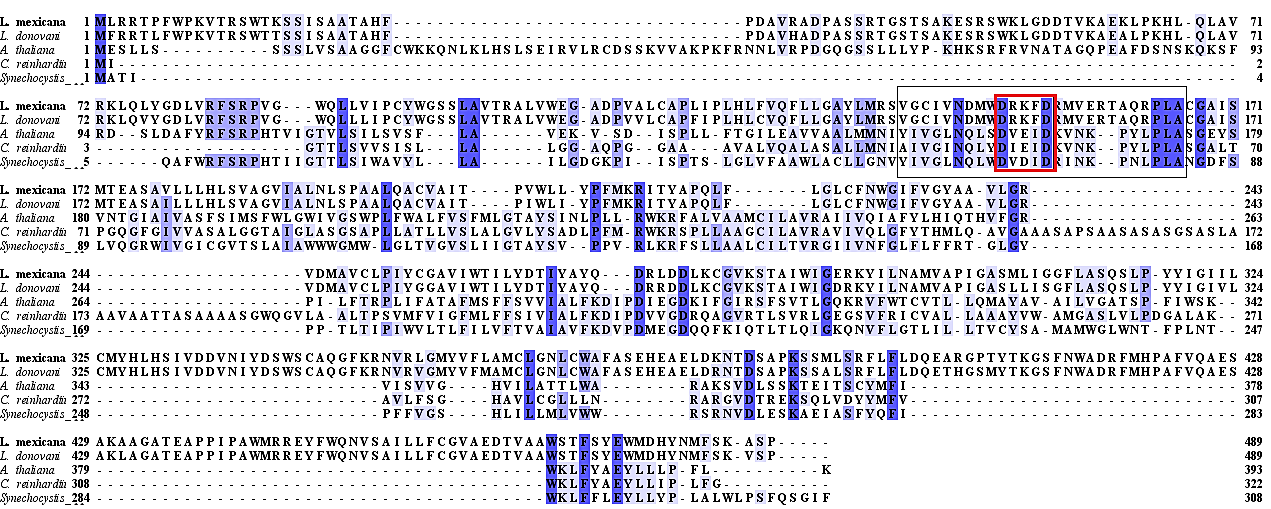


**FIGURE S3**

*****

*****
